# A Front Line Club Suggested

**Published:** 1918-12-07

**Authors:** J. F. Donegan


### THE EDITOR'S LETTER-BOX.

[Correspondence on all subjects is invited, but we cannot in any way be responsible for the opinions expressed by our correspondents, who must give their name and address as a guarantee of good faith, but not necessarily for publication. Correspondents are reminded that brevity of style and conciseness of statement greatly facilitate early insertion.]

### A Front Line Club Suggested.

*To the Editor of* The Hospital.

Sir,—I have been approached by many officers on the subject of which I write, and it remains to be seen whether the idea appeals to the profession generally. There are, as you know, different varieties of work done by medical officers, from service with local rank in their local towns or cities, to work at the Front with battalions and field ambulances. The Prime Minister, in eulogising the profession, alluded to the manner in which its leading members came forward and gave up their lucrative practices. These professional experts were almost invariably given the rank of colonel or major-general, and their zone of operations was chiefly confined to the base of operations. On the other hand, how many medical men came forward voluntarily, and, though not the leading members of the profession, left their families to exist as best they could and their practices to go to the dogs! How many of these gallant men have left their limbs behind them rotting in foreign countries, and how many, alas! have remained with their limbs—never to see their native land!

Now that peace is near the idea is that a sort of club should be formed, named "The Front Line Club," with the usual annual dinner, and be confined to medical officers who have served in the present campaign as regimental M.O.s and in field ambulances. Of course, this suggestion can be modified, and an officer who served as stated and also on the line of communications will be entitled to membership by virtue of his former position. It also goes without saying that the battalion or field ambulance with which the officer was employed must have been located otherwise than at the base of operations or line of communications. As regards Presidency, the present Director-General of the Army Medical Service would be well qualified, having himself been a field ambulance commander, and there may be many other senior officers of the Army Medical Services whose services have extended to the zone of actual hostilities.

The whole idea is at present only a suggestion, and if it appeals to the members of the profession a letter directed to Lt.-Colonel J. F. Donegan, C.B., c/o Holt and Co., 3 Whitehall Place, will be replied to and any suggestion recorded.

It is proposed that officers of the Royal Navy who served on ships which engaged the enemy, permanent R.A.M.C. and I.M.S. officers duly qualified as described, should be included, also officers of the Colonial Medical Services together with members of the medical profession who served with combatant ranks.—I remain yours faithfully,

J. F. Donegan, Lt.-Colonel R.A.M.C. (R.).

